# Changes in Violence and Clinical Distress Among Men in Individual Psychotherapy for Violence Against Their Female Partner: An Explorative Study

**DOI:** 10.3389/fpsyg.2021.710294

**Published:** 2021-07-23

**Authors:** Ingunn Rangul Askeland, Marianne Skogbrott Birkeland, Bente Lømo, Odd Arne Tjersland

**Affiliations:** ^1^Norwegian Centre for Violence and Traumatic Stress Studies, NKVTS, Oslo, Norway; ^2^Alternative to Violence-ATV, Oslo, Norway; ^3^Department of Psychology, Faculty of Social Sciences, University of Oslo, Oslo, Norway

**Keywords:** individual psychotherapy, partner violence, recidivism, treatment outcome, clinical distress

## Abstract

Most interventions for men who have acted violently toward their partner have been conducted as group interventions within a criminal justice context. Therefore, few studies have examined individual psychotherapy and how such interventions may reduce partner violence. In this study, we aimed to describe changes in violence, and changes in clinical distress in men undergoing individual psychotherapy targeting their use of partner violence, at a clinic organized within a psychosocial health care context. This is a naturalistic prospective study of men voluntarily receiving individual psychotherapy for their use of violence against their female partner. Participants were 84 male clients, and data on their use of physical violence, physical controlling violence, property violence and psychological violence were collected pretreatment, posttreatment and at follow-up 1.5 years after treatment from both the men, and their partners (*n* = 58). The percentage of use of all types of violence during a *typical month the last year* decreased from pretreatment to follow-up, according to both the men, and their partners. Over the course of treatment, use of all types of self-reported violence during *the last month* was reduced, however, this was only partially confirmed by their partners. Number of sessions was associated with a lower risk of having used physical and physically controlling violence 1.5 years after treatment. Alcohol abuse or dependency, or qualifying for one or more psychiatric diagnoses, were not associated with levels or change in use of violence. On average, the men's clinical distress declined over the course of psychotherapy. The findings suggest that individual psychotherapy may be a promising and worthwhile intervention for intimate partner violence. Studies with more elaborate designs are needed to identify the core mechanisms of psychotherapy for violence, and to corroborate these results with higher levels of evidence.

## Introduction

Male-to-female intimate partner violence (IPV) has extensive immediate and long-term negative effects on women‘s physical, mental, and sexual health (Loxton et al., 2017; Bacchus et al., 2018), and extorts high social and economic costs for both affected family members and societies (Krug et al., 2002; Hines et al., 2012). Worldwide, about one third of all women who have been in a relationship report having experienced one type of physical or sexual violence from their partner (World Health Organization, 2021). In Norway, one of the Scandinavian countries, all forms of violence are prohibited, including all use of corporal punishment, sexual and psychological abuse. Norway has a penal provision specifically concerning family violence and a general cultural attitude against violence in a family context. Nonetheless, in a national study, it was found that 16.3% men and 14.4% women reported exposure to “less severe” physical violence from a partner, and 9.2% women and 1.9% men reported exposure to “severe” physical violence (Thoresen and Hjemdal, 2014).

Over the years various treatment approaches based on different theoretical frameworks, settings and formats have been developed in an effort to stop and prevent partner violence, especially male-to-female IPV. The majority of these interventions have been based within a criminal justice context, where men convicted of domestic violence are mandated to attend a group-based batterer intervention program (e.g., Eckhardt et al., 2013; Wilson et al., 2021), constituting a combination of legal sanctions and therapeutic interventions. Although these programs vary in their methodology and stated goals, the majority have their origins in the Duluth model grounded in a sociocultural feministic framework evolving around men's attitudes and use of power and control (Pence and Paymar, 1993; Pence, 2002; Gondolf, 2012). More recent interventions include cognitive behavioral therapy (CBT), or programs with a mix of CBT and psychoeducational feminist components. Studies on such group-based interventions within a judicial context have shown some promising but partly equivocal results. While some reviews have found positive significant, but modest mean effects sizes (e.g., Eckhardt et al., 2013; Babcock et al., 2016), others have concluded that the effects of such programs seems inconclusive (e.g., Smedslund et al., 2011). In a recent meta-analysis, comparing pretreatment to posttreatment scores, a significant reduction in IPV were found when all interventions were pooled together. However, subgroup analysis found standardized treatment interventions based within a Duluth framework to generate mixed results (Karakurt et al., 2019). Another systematic review of court-mandated programs, found a modest but nonsignificant mean effect in favor of the program when outcome were based on official reports. However, the overall mean effect based on victim reports were either no difference or in favor of the no treatment condition, leaving the authors to determine that there is insufficient evidence to conclude that these programs are effective (Wilson et al., 2021). In sum, although the overall results are in a positive direction, it seems there is still some uncertainty as to the general efficacy of such court mandated programs, especially regarding the psychoeducational Duluth based models (Miller et al., 2013; Karakurt et al., 2019; Arce et al., 2020).

As the amount of research on men in IPV treatment programs has grown it has become evident that this group of men comprises a heterogeneous group. Researchers have identified men in treatment for IPV to vary across three common dimensions regarding: (a) the severity, frequency and type of violence used, (b) whether they use violence within the family only or extrafamilial, and (c) the degree of concurrent mental health disorders (Holtzworth-Munroe, 2000; Holtzworth-Munroe and Meehan, 2004; Johnson, 2008; Stoops et al., 2010). The levels of mental disorders and symptoms of mental health disorders among men in treatment for IPV has repeatedly been found to be much higher than in the general population (e.g., Rosenbaum and Leisring, 2003; Hamberger, 2008; Askeland and Heir, 2014). Furthermore, there is evidence that those who score on concurrent symptoms of mental health disorders tend to exhibit more severe or pervasive violence compared to those not meeting the criteria for a mental health disorder (Holtzworth-Munroe et al., 2000). In a study of men mandated to treatment, the researchers examined the association between symptoms of depression, PTSD, GAD, panic disorder, social phobia, and substance use disorders and the use of physical, psychological or sexual violence (Shorey et al., 2012). Firstly, the estimated prevalence rates of mental health problems, were found to be extremely high, with alcohol use disorders being the most prevalent. Secondly, all mental health problems, except sexual violence and panic disorder, were positively associated with IPV, and as the frequency of mental health problems increased, the frequency of IPV perpetration increased concurrently (Shorey et al., 2012). Others have found men in treatment for IPV classified with PTSD to be more frequently and severely aggressive than men without PTSD (e.g., Rosenbaum and Leisring, 2003; Creech et al., 2017).

Furthermore, studies have found that alcohol abuse in particular, to be highly associated with violence (Boden et al., 2012, 2013). Moreover, men in treatment for IPV typically report high levels of substance abuse (Dalton, 2001; Fals-Stewart, 2003). There is a general agreement that substance abuse increases both the frequency and severity of violence (Stuart et al., 2003; Murphy et al., 2005; Leonard and Quigley, 2017; Cafferky et al., 2018). Thus, we might expect that qualifying for a psychiatric disorder or an alcohol abuse or dependency diagnosis could predict poorer outcomes compared to not qualifying for a diagnosis.

The research displaying the diversity and successive variation in treatment needs among men in IPV treatment has inspired the development of programs targeting different subgroups of men or specific treatment needs, in an effort to enhance program outcome. Research on such more specifically targeted interventions have shown promising results (Holtzworth-Munroe, 2000, Cantos and O'Leary, 2014, Zarling et al., 2019). In the recent meta-analysis by Karakurt et al. (2019) it was for example found that incorporating substance abuse or trauma components to the interventions generated better results compared to programs that did not have these components. Several studies have also found positive results for applying strategies that tailor treatments to individual levels of readiness to change (e.g., Alexander and Morris, 2008, Levesque et al., 2008). Furthermore, the use of motivational interviewing to enhance the strength of the therapeutic alliance, and the individuals engagement in treatment, also seems to increase session attendance and subsequently reduce posttreatment violence (e.g., Taft et al., 2004; Taft and Murphy, 2007). Correspondingly, in a qualitative study of alliance formation between men in individual IPV treatment and their therapist, it was found that therapy processes with good outcomes were characterized by a working alliance that formulated the client‘s use of violence as his personal problem. This was opposed to what was found in unsuccessful cases, where use of violence was perceived more as “an unwanted behavior” but not linked to the client on a more personal and specific level (Lømo et al., 2019).

Regardless of the mechanisms undergirding the associations between violent behavior and different symptoms of mental health disorders, it seems plausible to enhance treatment outcome by addressing highly comorbid issues and tailor the treatment to the specific characteristics and therapeutic need of the individual man. This knowledge further implies the need of a more psychotherapeutically oriented approached, as opposed to an emphasis on psychoeducation. It may be productive to look at partner violence as having a complex and multifaceted etiology as a part of a complex symptom manifestation specific to each individual man. Overall, men in IPV treatment will most likely have comprehensive treatment needs, some of which may be similar while others may be very different. An individually adjusted treatment could apply a flexible exploration of the man's violent behavior and how his use of violence is related to his general psychological functioning, and potential problematic use of alcohol.

Based on the mean treatment length of 16 sessions in their meta-analysis, Babcock et al. (2004) proposed to compare effects of treatment programs shorter and longer than 16 sessions. Two meta-analyses found that longer treatment programs (>16 sessions) seemed more effective in reducing recidivism compared to short programs (<16 sessions) (Arce et al., 2020, Arias et al., 2013). Given the knowledge that there are great variations between these men it might be more productive to adjust program length to the individual accordingly. Furthermore, clinically significant change among men in treatment for IPV should expand beyond ending the violent behavior and explicitly include work to improve functioning in interpersonal relationships. Individual treatment has the advantage that it can be more flexible and tailored to the way in which the client's use of violence is related to his problematic relational patterns and mental health problems.

Accordingly, an outpatient clinic in Norway, Alternative to Violence, has developed an integrative form of violence-focused psychotherapy combining different therapeutic approaches and tools to meet the needs of the individual client. The psychotherapeutic practice at ATV is described similarly to how (Wampold and Imel, 2015, p. 37) define the term: a treatment based on psychological principles involving a trained therapist and a client who is seeking help, with assistance adapted and individualized for the particular client. However, the therapy will always focus specifically on the client‘s use of violence, his perception of his own violent behavior and ways to prevent further use of violence. Treatment is organized at a low threshold, aimed at reaching a wide variety of men using violence, and based on voluntary admittance. Approximately half of the men self-refer, with the remaining half are referred through public services (e.g., child protection services, social workers, family doctors, women's shelters, outpatient psychiatric services, emergency units, the police, prisons, etc.). Thus, treatment is not delivered as a part of a coordinated response within a criminal justice framework, but is based within a psychosocial health care context.

## The Aim of the Current Study

The main aim of this study was to explore changes in male-to-female partner violence and changes in clinical distress from pretreatment, to posttreatment and to follow-up 1.5 years after treatment, among men who acted violently against their female partner, and who voluntarily attended individual therapy offered at ATV. Furthermore, we have explored how intervention dose, concurrent symptoms of a mental health disorder, and alcohol abuse or dependency may affect outcome. More specifically, we aimed to do as follows:

1. Examine changes in male-to-female partner violent behavior from pretreatment to posttreatment and follow-up, as reported by male clients who received psychotherapy at ATV and as reported by male client's female partner;
2. Examine associations between covariates of the overall change of self-reported violence over the course of treatment;
3. Examine associations between the covariates; a) alcohol abuse or dependency, b) fulfilling the diagnostic criteria for one or more psychiatric diagnoses, and c) number of sessions, on self-reported pretreatment violence, rate of change, and violence 1.5 years after treatment; and
4. Examine changes in symptomatic and interpersonal distress from first treatment session to follow-up among men in treatment at ATV.

## Method

### Design and Procedure

This study is based on data from a prospective naturalistic process and outcome study of men going through treatment for their violent behavior toward their partner. Study participants were recruited from five outpatient clinics specializing in the treatment of violent behavior (ATV clinics). At intake, between January 2010 and July 2011, all male clients were interviewed face to face at their local ATV office by clinical psychologists with substantial clinical experience with the treatment of men who use violence. Data on violent behavior were obtained through a self-report questionnaire completed by male clients both pretreatment and at posttreatment. After the initial assessment of the male client, another clinical psychologist contacted the current or recent female partner and interviewed her by phone. The same procedure was followed at posttreatment. Finally, to examine whether the potential changes were lasting, the male clients and partners were interviewed by phone at follow-up 1.5 years after the end of treatment. Data on symptomatic and interpersonal distress were administered by a self-reported questionnaire to the male clients after the first treatment session, posttreatment and at follow-up. Data on symptoms of mental health disorders, including alcohol abuse and alcohol dependency, were collected as a clinical interview with the male client at pretreatment. All participants were verbally informed about the study, presented a written information letter and have given their written consent. The study was approved by the Regional Committees for Medical and Health Research Ethics and considered to be in compliance with privacy and ethical guidelines for medical research.

### Participants

**Figure 1:**
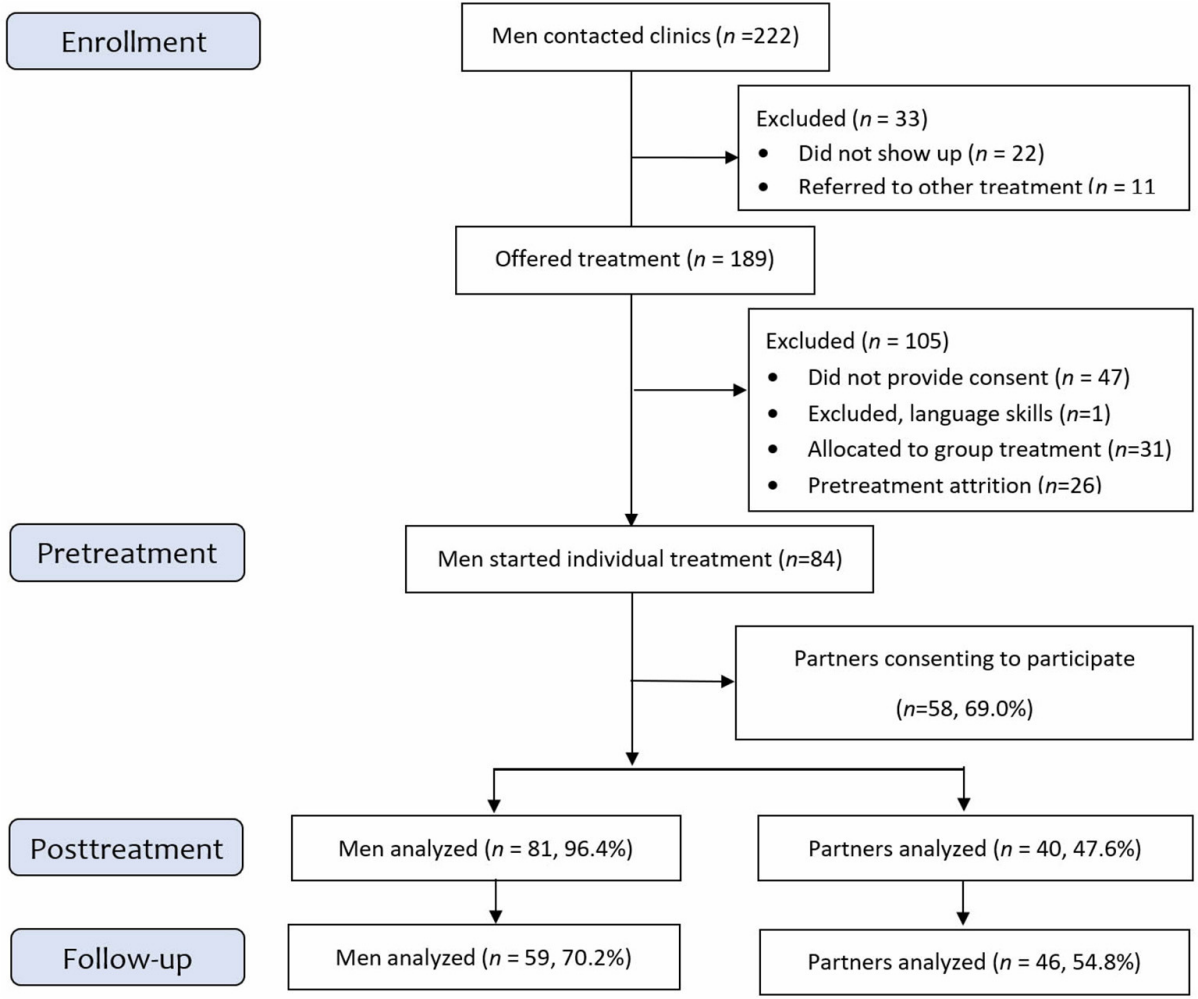
Flowcharts of participants.

The study population consisted of men voluntarily attending treatment for violence against their female partner. During the inclusion period, 222 men contacted one of the five ATV centers involved in the study (see Figure 1). Of these, 22 did not show up to the first assessment session. After the initial assessment, 11 men were referred to other treatments due to an acute need for psychiatric care or substance abuse rehabilitation. Of the 189 who were offered treatment at an ATV clinic, 47 did not provide consent to participate in the study, one was excluded from the study due to lack of Norwegian language skills, and 26 men did not show up for their first treatment session. A total of 115 men started treatment, of whom 31 were assigned to group therapy and 84 to individual therapy.

This study comprised 84 men who attended individual therapy. All the participants had used at least one type of violence against a current or recent partner. Their ages ranged from 19 to 72 years (mean age 37 years, SD = 10.4), 85.7% defined themselves as mainly having a Norwegian ethnic affiliation, 70.2% were either employed or studying, 61.9% were married or cohabitants, 70.2% lived with or had regular contact with their children (under the age of 18). A total of 59.5% had previously been in contact with the police in relation to a violent episode, 46.4% had been charged for a violent crime, 26.2% had previously been convicted for a violent crime, and 32.1% had been convicted for another crime. Of the convictions for a violent crime, 27.3% (*n* = 6) were against a partner/former partner, 54.6% (*n* = 12) were against strangers, 13.6% (*n* = 3) were against friends or acquaintances and 4.6% (*n* = 1) were against a police officer. The majority of the sample met the diagnostic criteria for at least one ongoing psychiatric disorder at the intake interview, measured by a structured clinical interview, the Norwegian version of Mini International Neuropsychiatric Interview (MINI), version 5.0.0 (Leiknes et al., 1992–2009). The distribution was as follows: major depressive disorder (*n* = 32, 38.1%), dysthymic disorder (*n* = 7, 8.3%), manic/hypomanic episode (*n* = 3, 3.6%), panic disorder (*n* = 2, 2.4%), agoraphobia (*n* = 12, 14,3%), generalized anxiety disorder (*n* = 5, 6.0%), social anxiety disorder (*n* = 10, 11.9%), obsessive-compulsive disorder (*n* = 7, 8.3%) post-traumatic stress disorder (*n* = 21, 25.0%), alcohol dependence (*n* = 16, 19.0%), alcohol abuse (*n* = 17, 20.2%), drug dependency (*n* = 4, 4.8%), drug misuse (*n* = 3, 3.6%), psychotic disorder (*n* = 0), anorexia/bulimia (*n* = 0), antisocial personality disorder (*n* = 21, 25.0%). Suicidality (*n* = 43, 51.2%): levels of suicidality- low (*n* = 25, 58.1%),—moderate (*n* = 7, 16.3%),—high (*n* = 11, 25.6%).

Due to ethical considerations demographic data on the 58 partners who agreed to participate in the study, were not collected.

### Psychotherapists

There were 15 therapists involved in the study, i.e., seven women and eight men. All of them were licensed clinical psychologists, with an average of 7.4 years (range 1–23) of experience with IPV therapy and an average of 9.4 years (range 3–23) of experience as a clinical psychologist. Based on a scale from 0 to 5 (0 being “not at all” to 5 being “very much”), all the therapists were asked to rank their degree of influence from the following theoretical orientations: analytic/psychodynamic, behavioral, cognitive, humanistic, systemic and other. Nine therapists contributed with information on theoretical orientation. Applying a definition of “moderately to strongly influenced by,” based on scores of 3, 4, or 5, the most frequently endorsed theoretical orientations were as follows: cognitive and humanistic (eight therapists), systemic (six therapists), and analytic/psychodynamic, behavioral or other theoretical orientation (three therapists). Except for one therapist who reported being moderately or strongly influenced by just two orientations, all the other therapists reported that they were moderately to strongly influenced by at least three different theoretical orientations. Four therapists claimed to be influenced at this level by four or five different orientations. Consequently, all therapists can be considered integrative and influenced by a wide range of different theoretical orientations.

### Violence-Focused Integrative Psychotherapy at ATV

There are 15 ATV clinics in Norway for adults who use violence against either partner or partner and children. The overall goal of the therapy is that the clients stop using all forms of violence and develop healthy and respectful ways of relating to others. Within a psychotherapeutic setting a therapist is obliged to conduct a thorough assessment and use their clinical expertise to determine if and how treatment should be offered.

The most dominant IPV intervention standard is the gender-based power and control model (e.g., Gondolf, 2012). Work within such a framework will emphasize men's patriarchal ideology and challenge power-and-control-based socialization patterns. The more psychological or intrapersonal models will emphasize different aspects of the person's intrapersonal functioning, often based on principles from CBT. Therapeutic models within such a framework will often extend their approach to accentuate such things as trauma related symptoms and information processing deficits (e.g., Taft et al., 2016a,b), insecure attachment styles (e.g., Dutton et al., 1994; Dutton and White, 2012), emotion regulation difficulties (e.g., Pascual-Leone et al., 2011), and concurrent substance abuse (e.g., Stuart, 2005, Stuart et al., 2006, Easton et al., 2007, Klostermann et al., 2010). The described therapeutic approach at ATV does not build on one theoretical model, as one single model does not seem to capture the complexity of IPV. In such a perspective the role of gender, and how the man perceives himself as a man in relationship to his female partner, will be perceived as one part of IPV etiology, but will also emphasize other intrapersonal and contextual factors as important contributors to violence. This is a form of assimilative integration (Stricker and Gold, 2003) that allows the therapist to use those approaches that seem useful, nevertheless always in the frame of addressing the client's use of violence.

Thematically, the ATV model is similar to what has been described in other IPV literature, however the difference may be more related to *how* one works with the different themes. While several IPV interventions seem to emphasize psychoeducational aspect, this is not the case in treatment at ATV. The therapeutic work does not accentuate psychoeducational aspects of therapy, but rather the exploration of the client's specific and personal pathways in how he creates his own violence.

The overarching therapeutic model comprises four essential and overarching themes that the therapists in different ways should explore in their encounter with the client, across treatment modalities. The themes are:—the client's *use of violence*,—his *sense of responsibility*,—*psychological connections* (how violence relates to how they think, feel, their intentions, and how it relates to their personal history), and—*consequences of violence* (Råkil, 2006; Askeland and Råkil, 2017; Askeland et al., 2020). The four essential themes are not to be understood as phases, rather, these themes represent the therapist‘s main areas to explore and navigate by in different ways throughout the entire therapy process. An integrative perspective allows a broad exploration of the specific mechanisms behind each man's violent behavior. Thus, the therapeutic work at ATV is evolving around the abovementioned themes and explores the individual man's violence from a more meta-theoretical perspective, assisting him in exploring himself and guiding him in a personal development. The therapeutic work may vary according to what seem to be important therapeutic pathways in helping the client stop their violent behavior. This may be work on developing their relational competence, exploring and challenging problematic thoughts and beliefs, and enhancing their ability to understand and cope with their own emotional states and their capacity to mentalize both themselves, partners and children.

Attendance is voluntary and not time-limited. Based on a thorough assessment, partner information, and the therapist's evaluation of progress and the therapeutic needs of each client, treatment length is adjusted over the course of treatment. All the partners are invited to an individual session with a therapist. This session allows the partners to gain information about the treatment, and guidance about other help and support agencies. Furthermore, it gives the therapist essential information on the violence and safety for partners and children. Partners who want help related to their experiences of violence are offered treatment at the clinic. For more detailed descriptions on therapeutic work at ATV see: (Lømo, 2018; Lømo et al., 2019; Askeland et al., 2020).

### Measures

Violent behavior was measured with the Violence, Alcohol and Substance Abuse (VAS) questionnaire, a shortened version of the Violence Questionnaire (VQ) (Askeland and Heir, 2014, Strandmoen et al., 2016). The VAS questionnaire has 32 predefined descriptions of different violent behaviors, covering five different types of violence: physical violence against current or last partner—(e.g., punch against body, punch against head, kick, choke); physically controlling violence (e.g., shake, shove, tug, wrench arm); property violence—(e.g., hit walls, throw objects, destroy objects); psychological violence—(e.g., threats of violence, use threats to get your will, interrogate, name calling, make fun of/humiliate her); and sexual violence—(e.g., force intercourse, other forced sexual activity, sexual humiliation of partner). All men were informed about the definitions of the different types of behaviors and asked to specify how many times they had used the described behaviors or equivalent behaviors during the “last month” and during a “typical month” in the past year. This approach was taken since some violent behavior might be low frequency; thus, asking about the “last month” might be less suitable for identifying such violence. The low frequency types of violence typically reported in a “typical month” would be coded as a positive score if there was at least one incident within the previous year. Furthermore, the last month prior to starting psychotherapy for violence against their partner may not be a representative month.

#### Clinical Distress

Treatment includes therapeutic work on clients' more general psychological functioning; thus, we wanted to measure potential changes in clients' relational functioning and symptoms of distress. To accomplish this, we applied the Outcome Questionnaire-45 (OQ-45) (Lambert et al., 2004). The OQ-45 addresses the symptoms most commonly present across psychiatric disorders relevant to interpersonal functioning (Beckstead et al., 2003). The OQ-45 is a 45-item self-report instrument used to let clients rate their mental health distress and functioning on a five-point Likert scale and consists of three subscales: (a) symptom distress, (b) interpersonal functioning, and (c) social role (Beckstead et al., 2003).

#### Symptoms of Mental Health Disorders

To measure symptoms of mental health disorders and whether the men met the diagnostic criteria for an ongoing psychiatric disorder, we administered the Mini International Neuropsychiatric Interview [Mini International Neuropsychiatric Interview [MINI], Lecrubier et al., 1997]. MINI is a brief and valid structured diagnostic interview that covers 16 axis I disorders and the axis II antisocial personality disorder according to the Diagnostic and Statistical Manual of Mental Disorders, Fourth Edition (DSM-IV) criteria. We used the Norwegian version of Mini International Neuropsychiatric Interview (MINI), version 5.0.0 (Leiknes et al., 1992–2009).

#### Measures Collected but Not Included in This Study

At the intake session, clients were interviewed on their trauma experiences with TEC [Traumatic Experiences Checklist [TEC]; Nijenhuis et al., 2002], and the working alliance inventory (WAI-SR, Hatcher and Gillapsy, 2006).

### Data Analyses

Changes in proportions of self-reported and partner-reported violence across specific time points were tested with McNemar's exact binomial tests. Change in self-reported violence over the general time course was described using latent categorical growth curve modeling (LGCM) (Lee et al., 2017). As the variables were ordinal, they were specified as categorical in Mplus. The observed categorical variables were transformed into latent continuous variables using the probit link function with the ML with robust standard errors (MLR) in Mplus. These were used as input in an unconditional latent growth curve model. Factor loadings for the slope factor were fixed to 0 (typical month during the last year), 1 (baseline month), 2 (last month at treatment end), and 3 (last month at follow-up 1.5 years after treatment end). Next, a conditional growth curve model with intercept and slopes regressed on the three covariates (alcohol dependence or abuse, diagnosis, and number of sessions) were specified. Finally, we re-specified the conditional growth curve in such a way that the intercept reflected the endpoint, and we regressed this second intercept and slope set on the same covariates. Due to low number of participating partners, it was not possible to conduct this analysis with partner-reported violence.

Change in clinical distress between session 1 of treatment and follow-up were investigated with descriptive analysis, *t*-tests and McNemar's test. The analyses were done in IBM SPSS Statistics 26 (IBM Corp, 2019) and Mplus version 8.3 (Muthén and Muthén, 1998-2019).

### Missing Data

The total sample consisted of 84 men, of which 81 provided data at posttreatment and 59 participated at follow-up. Among the 84 participants, 59 (70.2%) provided data at all three time points, and 22 (26.2%) provided data at two of the time points, whereas three (3.6%) provided data at only one time point. To assess selective participation over time, participation was regressed on previous scores of each of the types of violence. Logistic regressions indicated that missing data were not significantly related to either of the measures of violence. In addition, of the 84 men, 57 (67.9%) had partners who provided data pretreatment, 40 who provided data at posttreatment and 46 who provided data at follow-up. To assess the possible pretreatment selective participation of partners, the pre-treatment participation of partners was regressed on the scores of the men's pretreatment self-reported violence. The logistic regressions indicated that missing data from partners did not significantly relate to any of the measures of violence.

## Results

### Self-Reported and Partner-Reported Violence at Each Time Point

Both the men and the partners reported that most common type of self-reported violence was psychological violence, followed by property violence and physically controlling violence (see Table 1). Self-reported incidences of sexual violence were very low (*n* < 5) and are therefore not presented. A greater percentage of men reported to have used violence during a typical month the last year, compared to the percentage that used violence the last month prior to treatment. For example, whereas 39.3% of the men and 44.8% of the partners reported physical violence during a typical month in the last year, only 13.1% of the men and 17.5% of the partners reported physical violence the month prior to the treatment. Thus, it seems like the last month prior to starting psychotherapy may not be a representative month.

**Table 1:** Proportions (*n*) of men self-reporting at least one incidence of male to female partner violence, and partner-reports of at least one incidence of male to female partner violence.

	**During a typical month during the last year**			**During the last month**					**Typical and last month**
	**Pre**	**Follow-up^a^**	**Pre–follow *p*-value^b^**	**Pre**	**Post**	**Follow-up^a^**	**Pre–post *p*-value^b^**	**Post–follow-up *p*-value^b^**	**Pre *p*-value^c^**
	***% (n)***	***% (n)***		***% (n)***	***% (n)***	***% (n)***			
Self-reported violence	*(n = 84)*	*(n = 59)*		*(n = 84)*	*(n = 81)*	*(n = 59)*			
Physical violence	39.3 (33)	3.4 (2)	<0.001	13.1 (11)	1.2 (1)	3.4 (2)	0.002	1.000	<0.001
Physically controlling violence	53.6 (45)	17.2 (10)	<0.001	23.8 (20)	12.3 (10)	6.8 (4)	0.035	0.453	<0.001
Property violence	66.3 (55)	22.0 (13)	<0.001	23.8 (20)	17.7 (14)	11.9 (7)	0.332	0.607	<0.001
Psychological violence	78.6 (66)	29.3 (17)	<0.001	56.0 (47)	29.6 (24)	15.5 (9)	0.001	0.004	<0.001
Partner-reported violence	*(n = 58)*	*(n = 46)*		*(n = 58)*	*(n = 40)*	*(n = 46)*			
Physical violence	44.8 (26)	10.9 (5)	<0.001	17.5 (10)	7.5 (3)	0.0 (0)	0.435	0.250	0.003
Physically controlling violence	57.9 (33)	13.0 (6)	<0.001	24.1 (14)	17.5 (7)	4.3 (2)	0.549	0.063	<0.001
Property violence	73.7 (42)	19.6 (9)	<0.001	34.5 (20)	25.6 (10)	8.9 (4)	0.678	0.016	<0.001
Psychological violence	92.9 (52)	43.5 (20)	<0.001	70.2 (40)	56.4 (22)	34.8 (16)	0.227	0.065	0.001

a *Follow-up, 1.5 years after posttreatment*.

b *McNemar's exact binomial test*.

c *McNemar's exact binomial test of any reported violence during a typical month during the last year, and during the last month, at pretreatment*.

The percentage of men who reported having used physical violence, physically controlling violence, and psychological violence during the last month decreased significantly from pre-treatment to post-treatment. No significant reduction in property violence was found. Although the numbers seem to indicate that the percentages of partner-reported violence during the last month seem to decrease from pretreatment to post-treatment, the results show no significant differences. Of the 26 men who reported to have used physical violence during a typical month at pretreatment and who responded at follow up, 100% reported they had not used physical violence during a typical month at follow up. Corresponding percentages for physical controlling violence, material violence and psychological violence were: 74.7, 77.1, and 62.1%. Regarding the partners; of the 26 partners who reported that the men had used physical violence during a typical month at pretreatment and who responded at follow up, 80.8% reported that he had not used physical violence during a typical month at follow up. Corresponding percentages for physical controlling violence, material violence and psychological violence were: 79.4, 72.3, and 49.2%.

From post-treatment to follow up, few differences were found—with two exceptions. The percentage of men reporting having used psychological violence during the last month was further significantly reduced, and the percentage of partner-reported property violence during the last month decreased significantly. However, in both men and partners, the percentage reporting violence during a typical month during the last year declined significantly from pre-treatment to follow-up.

### Covariates of Overall Change in Self-Reported Violence Over the Course of Treatment

To examine associations between covariates of the overall change of self-reported violence over the course treatment, we specified a linear growth curve of each type of violence. The unstandardized estimates of means of variances in intercepts and slopes of self-reported violence and are presented in Table 2. The means of slopes show that on average, all types of self-reported violence decreased across time.

**Table 2:** Unstandardized estimates (Est) and standard errors (SE) of means and variances of parameters of linear growth models of self-reported violence^a^ (*n* = 84).

	**Intercept mean**			**Slope mean**			**Intercept variance**			**Slope variance**		
	**Est**	**SE**	***p***	**Est**	**SE**	***p***	**Est**	**SE**	***p***	**Est**	**SE**	***p***
Physical violence against current or last partner	0.00	0.00	-	−1.34	0.29	0.000	0.28	0.81	0.638	0.40	0.31	0.198
Physically controlling violence	0.00	0.00	-	−1.33	−0.35	0.000	2.99	1.93	0.120	1.09	0.73	0.135
Property violence	0.00	0.00	-	−0.73	0.18	0.000	0.55	0.52	0.290	0.12	0.16	0.442
Psychological violence	0.00	0.00	-	−1.10	0.23	0.000	2.11	1.29	0.101	0.60	0.39	0.121

a *Violence is measured at four time points; typical month last year prior to treatment, last month pre-treatment, last month post-treatment, and last month follow-up*.

The associations between the covariates (alcohol dependence or abuse, qualifying for one or more psychiatric diagnoses, and number of sessions) and self-reported violence the typical month the last year, rate of change, at follow up can be seen in Table 3. We found no significant associations between alcohol dependence or abuse, qualifying for one or more psychiatric diagnoses, and number of sessions, and whether the men used violence at a typical month. Number of sessions was associated with a faster decline of physical violence, and lower physical violence and physically controlling behavior at follow-up. In addition, alcohol abuse or dependency was associated with lower property violence at follow up.

**Table 3:** Standardized estimates and standard errors of associations between intercepts and slope of self-reported violence, and covariates (*n* = 84).

	**Intercept start (typical)**			**Slope**			**Intercept endpoint (follow-up)**		
	**Est**	**SE**	***p***	**Est**	**SE**	***p***	**Est**	**SE**	***p***
**PHYSICAL VIOLENCE**									
Alcohol abuse or dependency^a^	0.62	0.32	0.056	−0.05	0.21	0.823	0.13	0.16	0.426
Diagnosis^a^	−0.01	0.24	0.981	−0.20	0.17	0.260	−0.20	0.17	0.218
Number of sessions	0.27	0.24	0.263	−0.81	0.13	<0.001	−0.77	0.12	<0.001
**PHYSICALLY CONTROLLING VIOLENCE**									
Alcohol abuse or dependency^a^	0.30	0.16	0.064	−0.22	0.22	0.322	−0.05	0.21	0.800
Diagnosis^a^	−0.18	0.17	0.307	−0.02	0.18	0.901	−0.14	0.17	0.391
Number of sessions	−0.10	0.17	0.555	−0.26	0.19	0.169	−0.37	0.19	0.048
**PROPERTY VIOLENCE**									
Alcohol abuse or dependency^a^	−0.19	0.23	0.388	−0.36	0.33	0.268	−0.51	0.20	0.012
Diagnosis^a^	−0.10	0.22	0.674	0.07	0.25	0.674	0.01	0.19	0.958
Number of sessions	0.21	0.19	0.258	−0.36	0.27	0.186	−0.23	0.27	0.186
**PSYCHOLOGICAL VIOLENCE**									
Alcohol abuse or dependency^a^	−0.03	0.18	0.880	−0.03	0.21	0.886	−0.07	0.19	0.736
Diagnosis^a^	0.14	0.19	0.484	−0.09	0.21	0.684	0.01	0.18	0.977
Number of sessions	−0.09	0.15	0.537	−0.14	0.17	0.404	−0.27	0.16	0.083

a *Yes vs. no*.

### Changes in Mental Distress

Table 4 shows that the mean levels of mental distress as measured by the OQ-45, decreased significantly from the first session to follow-up. This is evident for all the subscales of OQ-45. Additionally, the percentage of men reporting clinically significant levels of mental distress decreased significantly, from 60.5% at session 1 to 24.6% at follow-up.

**Table 4:** Mean levels, and percentages of men scoring above cutoff (>63) on clinical distress (OQ-45) at session one and at follow-up.

	**Session 1**	**Follow-up**	***p*-value^a^**	**Cohen's d**
*N*	81	57		
OQ-45 total: Mean (SD)	71.3 (25.1)	52.8 (25.2)	<0.001	−0.73
OQ-45 total: % (number) over cutoff	60.5 (49)	24.6 (14)	<0.001	
OQ-45: Symptom distress	40.2 (16.0)	30.2 (15.6)	<0.001	−0.63
OQ-45: Interpersonal functioning	17.2 (6.4)	12.7 (5.9)	<0.001	−0.72
OQ-45: Social role	11.2 (5.1)	8.9 (5.4)	0.001	−0.44

a *T-test (continuous variables) or McNemar's exact binomial test (categorical variables)*.

## Discussion

The main results of this study are that: (a) Self-reported violence declined throughout psychotherapy, (b) this was partially confirmed by partners, (c) the more sessions the men attended, the lower probability of using physical violence 1.5 after end of psychotherapy, (d) psychological violence may be more difficult to change, and e) most men reported less clinical distress 1.5 years after end of psychotherapy.

### Self-Reported Violence

Generally, we found a substantial decrease from pretreatment to after treatment, especially for the two types of physical violence. The overall change in self-reported violence also showed that the men had different reports of violence at pretreatment, and changed at different rates, but on average, all types of self-reported violence decreased across time. All types of self-reported violence, during a *typical month*, decreased significantly from pretreatment to follow-up. As some violent behavior could be low in frequency it might not be identified when asked within a narrow time frame. This would typically be true for the types of physical violence. Reaching out to an ATV clinic could perhaps have a positive influence on the man's awareness of own violent behavior. As the month pretreatment covers the time frame in which the man might have taken this first step in the direction of change, this month risk presenting an underestimation of the violence.

Regarding the overall rates of physical violence after treatment, it seemed to be rather low compared to what is typically found in outcome studies of IPV interventions. For example, in a recent outcome study in which half of the participants attended individual treatment, almost 50% of the total sample used some form of physical violence during the 6 months after treatment (Murphy et al., 2017). However, it should be noted that this rate was a composite measure including reports from clients and their partner and is therefore not directly comparable with our results.

At follow-up, there is still both self-reported and partner-reported violence. Even if the main goal of any intervention for IPV is to attain a zero-violence outcome on all forms of violence, our findings suggest a considerable positive change from pretreatment to after treatment and that this change was sustained until at least 1.5 years after treatment. We have not been able to identify any outcome studies based on voluntarily attending clients in individual treatment within a comparable health care context. Thus, it is difficult to compare these results to what has typically been found in similar types of outcome studies. However, studies of men in mandated batterer treatment programs, comparing rates of violence before and after treatment tends to find the recidivism rates of physical re-assaults to be approximately one-third (Davis and Taylor, 1999). In sum, even if there were still reports of violence, our findings suggest relatively low recidivism rates, which is especially evident for the two forms of physical violence. These results are promising regarding offering individual psychotherapy to similar groups of men to reduce violent behavior.

### Partner-Reported Violence

The partner-reports did only partially confirm the changes reported by the male clients. Although all types of partner-reported violence, during a *typical month*, decreased significantly from pretreatment to follow-up, when asked about the *last month*, the differences were not significant.

In general, more partners reported incidences of violence compared to the men; this was especially true for psychological violence. This finding is in line with earlier studies that show that partners tend to report more violence than what is found within self-reports of violence (e.g., Armstrong et al., 2002, Strandmoen et al., 2016). Partner reports are generally perceived as the “gold standard” in outcome evaluations of treatment for intimate partner-violent men (Bennett and Williams, 2001). On a theoretical level, it may be fruitful to explore the differences between self-reported and partner-reported violence. Whereas, physical violence contains observable behaviors, psychological violence consists of explicitly threatening or humiliating actions as well as behaviors that are more open to different interpretations. A natural consequence of having been exposed to violence by a partner is that one may feel unsecure for a long time. One may feel and thus, report being subjected to psychological violence by one's partner because one does not feel safe and may never feel safe in that relationship. This is a natural consequence of being violated, traumatized and experiencing a profound breach of trust; thus, some partners may report being subjected to psychological violence due to their feelings of being unsafe. This resonates with clinical experiences where clients often need help to understand how their partners are affected by their violence. A part of developing new relational skills and enhancing empathic understanding is assisting clients to understand and adequately respond to how their violence has affected their partners and that even if they feel that they have changed, it may take a long time before their partners perceive them as non-violent and feel safe in a relationship with them.

### Covariates and Self-Reported Violence

We found no associations between the covariates alcohol abuse or dependency, fulfilling the diagnostic criteria for one or more psychiatric diagnoses, and the rates of changes, or violence 1.5 years after treatment.

This could indicate that treatment at ATV may be equally well-suited for men with symptoms of mental health disorders, or an alcohol abuse or dependency problem, and those without such symptoms. Although there are comprehensive theoretical and clinical arguments for integrating interventions for IPV and substance abuse, there is still little empirical evidence indicating that such approaches enhances violence reduction (Stephens-Lewis et al., 2019).

However, the more sessions the men attended, the lower probability of using physical and physically controlling violence 1.5 years after end of psychotherapy. Thus, it seems that obtaining change requires a substantial numbers of sessions. This result is as expected. As violet behavior is a complex phenomenon, expanding far beyond simply being an attitudinal or behavioral problem, one could expect that the client would need a substantial numbers of session to develop as a safe and caring partner and father. The numbers of sessions were also found to be associated with a faster decline in physical violence. This is consistent with our other findings, it seems like over the course of treatment, physical violence appears to be declining more rapidly compared to psychological violence.

### Psychological Violence

Psychological violence was reported most frequently, at all three time-points. At pretreatment almost 93% of the partners and almost 79% of the male clients conveyed use of psychological violence during a *typical month* in the past year. This implies that psychological violence is a profound and extensive feature among this group of men in treatment for IPV. Whereas, the two forms of physical violence seemed to be considerably reduced, a substantial number of the men still reported psychological violence at the end of treatment. This is in line with earlier studies that found psychological abuse to have a higher base rate of occurrence than physical abuse (Murphy and Hoover, 1999, O'Leary, 1999, Strandmoen et al., 2016) and that psychological violence may continue even when physical violence decreases or stops (Hamberger and Hastings, 1988). Stopping the use of physical violence may be perceived as a more purely behavioral or *quantitative* change of *not* doing a physical act; thus, one can expect that it takes less time to achieve change in physical violence compared to psychological violence. Changes in psychological violence may require deeper and more profound *qualitative* change sin such as relational competence, and a more enhanced control over cognitive and emotional dysfunctional schema. This may also explain the observed further reduction in psychological violence between posttreatment and follow-up. Additionally, compared to physical violence, psychological violence includes more ambiguous and less tangible behaviors. Thus, it may take longer time for men to identify and stop using these forms of behaviors and develop new strategies in relation to their partner. This is in line with findings from a qualitative study of some of the long term, good outcome cases drawn from the same sample as the current (Lømo, 2018). The analysis revealed that the therapist and the client repeatedly engaged in exploring the client's experiences of having to cope with an ≪annoying≫ partner. This enduring therapeutic work, helped the client clarify his personal problematic patterns of being an intimate partner and father and gradually enhanced his capacity to take into account his partner's and children's state of mind (Lømo, 2018). Thus, psychological violence should be an important area to explore throughout the whole course of treatment.

### Clinical Distress

Our findings also suggest positive changes in clinical distress, particularly in interpersonal functioning, as the majority of the men reported less clinical distress 1.5 years after end of psychotherapy This finding is line with the goals of treatment, which emphasize that change should not only be to stop violent behavior but also to help clients deal with their difficulties, especially to enhance their relational skills and competence. According to the OQ-45 manual, a low score on this subscale suggests both the absence of interpersonal problems and satisfaction with the quality of intimate relationships (Lambert and Ogles, 2004). Moreover, enhanced functioning in interpersonal relations may be linked to our finding of an additional decline in the use of psychological violence found at follow-up. As men develop their capacity to regulate their own emotions and the ability to mentalize their partner's perspective, one can assume that they enhance their relational capacity and engage with their partner in more functional and respectful ways.

### Strengths and Limitations

This study applied a naturalistic design and was conducted within usual clinical practice; hence, the study has high ecological validity. Furthermore, we have applied a strict “zero tolerance” model for measuring change by using a dichotomous variable for measuring violence. Thus, we have minimized the possibility of overestimating the possible changes in levels of violent behavior. Also, all therapists were blind to the responses given by the clients.

The study included data from partners. As men using violence often tend to underreport the frequency, severity or impact of their violent behavior (Armstrong et al., 2002), it is crucial to include measures of partner-reported violence. This is also evident in a previous study on couple agreement on male-to-female violence based on data from pretreatment at ATV (Strandmoen et al., 2016). The current sample are included in the sample described in the prior study. Furthermore, looking at recidivism rates based on police reports is problematic, as rates of arrest are relatively infrequent compared to actual rates of partner violence. Additionally, by allowing a relatively long time period before follow-up measurement, we attained more reliable knowledge of long-term changes and whether changes found post-treatment endured or developed in a negative or positive direction.

Nonetheless, our preliminary evaluation applied pretest/posttest designs, which have limitations regarding how to interpret the results. This design does not allow for any firm conclusions regarding treatment effects. Additionally, the results found with this volunteer sample cannot automatically be generalized to a sample of men mandated to treatment within a juridical frame, typically reported in the literature of court-mandated samples from the USA or UK. Intuitively, these two populations may be different in several aspects, such as level of motivation and inequalities, such as different socioeconomic status. The ATV clinics have taken specific initiatives to reach out to minorities, such as non-Norwegian speaking populations, groups with different gender or sexual orientations and a diversity of ethnic and cultural minority groups. Although the sample comprises men from non-Norwegian ethnic and cultural backgrounds, all participants had to speak Norwegian. Treatment courses with interpreter were not included in the study. Furthermore, the study population consists of men in a heterosexual relationship. Thus, the results may not be applicable to the diversity of partner violent men in Norway.

Additionally, the sample size is relatively small; and there was a considerable degree of attrition; consequently, the analyses may be underpowered; thus, these results should be interpreted with caution. Another interesting future study could be to explore the associations between changes in interpersonal functioning and violence, especially psychological violence. Finally, the partners who no longer lived with the men at follow-up may have limited knowledge on changes in their violent behavior.

### Research Implications

More rigorously designed studies of this form of treatment are needed. This includes replicating the study with the same population and applying an experimental design. Furthermore, it is essential to include more research on what seems to work and for whom and to explore underlying mechanisms of change. It seems important to gain more knowledge on what seems to constitute the most potent elements in treatment and what therapeutic steps and characteristics appear to be important in achieving positive outcomes. Finally, including more sophisticated outcome measures, including potential changes in relationship quality, seems warranted.

### Clinical Implications

Our study suggests that it may be beneficial to offer individual violence-focused psychotherapy to men who have acted violently toward their partner. Most violence in intimate relationships will never be reported to the police, thus offering treatment at a low threshold may reach a larger group of men. Furthermore, such treatment allows for thorough clinical assessments and considerations related to whether treatment seems appropriate, plausible and ethically rightful. Individual treatment allows for flexible adjustments to clients' differences in motivation, cognitive function and specific challenges. Furthermore, a psychotherapeutic framework promotes the development of a strong working alliance, known to enhance treatment outcome, where the therapist and the client establish an empathic and productive alliance working together to reach reasonable and healthy treatment goals (Eckhardt et al., 2006, Wampold, 2015).

## Data Availability Statement

The raw data supporting the conclusions of this article will be made available by the authors, without undue reservation.

## Ethics Statement

The studies involving human participants were reviewed and approved by The Regional Committees for Medical and Health Research Ethics. The patients/participants provided their written informed consent to participate in this study.

## Author Contributions

All authors listed have made a substantial, direct and intellectual contribution to the work, and approved it for publication.

## Conflict of Interest

The authors declare that the research was conducted in the absence of any commercial or financial relationships that could be construed as a potential conflict of interest.

## Publisher's Note

All claims expressed in this article are solely those of the authors and do not necessarily represent those of their affiliated organizations, or those of the publisher, the editors and the reviewers. Any product that may be evaluated in this article, or claim that may be made by its manufacturer, is not guaranteed or endorsed by the publisher.
